# Systematic revision of the adeleid haemogregarines, with creation of *Bartazoon* n. g., reassignment of *Hepatozoon argantis* Garnham, 1954 to *Hemolivia*, and molecular data on *Hemolivia stellata*

**DOI:** 10.1051/parasite/2015031

**Published:** 2015-11-09

**Authors:** Grégory Karadjian, Jean-Marc Chavatte, Irène Landau

**Affiliations:** 1 UMR 7245 MCAM MNHN CNRS, Muséum National d’Histoire Naturelle 61 rue Buffon, CP 52 75231 Paris Cedex 05 France; 2 Malaria Reference Centre – National Public Health Laboratory, Ministry of Health 3 Biopolis Drive, Synapse #05-14/16 Singapore 138623

**Keywords:** *Hepatozoon*, *Bartazoon*, *Hemolivia*, *Karyolysus*, Life cycle, Systematics revision, Molecular data

## Abstract

Life cycles and molecular data for terrestrial haemogregarines are reviewed in this article. Collection material was re-examined: *Hepatozoon argantis* Garnham, 1954 in *Argas brumpti* was reassigned to *Hemolivia* as *Hemolivia argantis* (Garnham, 1954) n. comb.; parasite DNA was extracted from a tick crush on smear of an archived slide of *Hemolivia stellata* in *Amblyomma rotondatum*, then the 18S ssrRNA gene was amplified by PCR. A systematic revision of the group is proposed, based on biological life cycles and phylogenetic reconstruction. Four types of life cycles, based on parasite vector, vertebrate host and the characteristics of their development, are defined. We propose combining species, based on their biology, into four groups (types I, II, III and IV). The characters of each type are defined and associated with a type genus and a type species. The biological characters of each type are associated with a different genus and a type species. The phylogenetic reconstruction with sequences deposited in the databases and our own new sequence of *Hemolivia stellata* is consistent with this classification. The classification is as follows: Type I, *Hepatozoon* Miller, 1908, type species *H. perniciosum* Miller, 1908; Type II, *Karyolysus* Labbé, 1894, type species *K. lacertae* (Danilewsky, 1886) Reichenow, 1913; Type III *Hemolivia* Petit et al., 1990, type species *H. stellata*, Petit et al., 1990; and Type IV: *Bartazoon* n. g., type species *B. breinli* (Mackerras, 1960).

## Abstract (fr)

Une revue des cycles biologiques et des données moléculaires concernant les Hémogrégarines terrestres est effectuée. Du matériel de collection a été réexaminé : *Hepatozoon argantis* Garnham, 1954 chez *Argas brumpti* est reclassé dans *Hemolivia* comme *Hemolivia argantis* (Garnham, 1954) n. comb ; de l’ADN a été extrait d’un écrasement de tique sur lame, provenant d’une partie du matériel type d’*Hemolivia stellata* chez *Amblyoma rotondatum*, puis le gène 18S ssrRNA a été amplifié par PCR. Une révision systématique du groupe, basée sur les cycles biologiques et sur les reconstructions phylogénétiques est proposée. Quatre types de cycles biologiques sont définis, en fonction du vecteur, de l’hôte vertébré, des parasites et des caractéristiques de leurs développements. Nous proposons de rassembler les espèces en fonction de leur biologie en quatre groupes (types I, II, III, IV). Les caractères de chaque type sont définis et associés à un genre et à une espèce type. La reconstruction phylogénique faite à partir de séquences déposées dans les banques de données et notre nouvelle séquence *d’Hemolivia stellata* est compatible avec cette nouvelle classification. La classification est la suivante : Type I, *Hepatozoon* Miller, 1908, avec espèce-type *H. perniciosum* Miller, 1908 ; Type II, *Karyolysus* Labbé, 1894, avec espèce-type *K. lacertae* (Danilewsky, 1886) Reichenow, 1913 ; Type III *Hemolivia* Petit et al., 1990, avec espèce-type *H. stellata*, Petit et al., 1990 ; Type IV : *Bartazoon* n. g., avec espèce-type *B. breinli* (Mackerras, 1960) n. comb.

## Introduction

The haemogregarines form a group of particularly diverse heteroxenous adeleid coccidia parasites which have exploited all environments, terrestrial or aquatic, and become adapted to numerous vertebrate hosts, i.e. chelonians, crocodiles and other reptiles, amphibians, fishes and many mammals.

(*i*) *In the aquatic environment* – the transmission of parasites is obligatorily achieved either by predation between vertebrates [38, 70] or through vectors in close contact with the vertebrate hosts.

For example, in the wild, an *Eimeria* of fish may be transmitted from fish to fish by cannibalism or via a paratenic host such as a shrimp [38, 70] but not, in natural conditions, by shedding oocysts in the water where they would be immediately diluted.

The haemogregarines of aquatic hosts are transmitted by leeches or by arthropods in which the sexual part of the cycle develops. The sporogony of *Haemogregarina stepanowi* Danilewsky, 1885 [14] develops in the leech which transmits the infection when feeding on the turtle [60]. The oocysts in the leech are asporate and produce free sporozoites which are inoculated to the turtle. In some vectors of the haemogregarines of fish, a further stage develops from the sporogony: a merogony, in the leech for *Cyrilia* Lainson, 1981 [36], in the isopod for *Desseria* Siddall, 1995 [65]; the vertebrate host would become infected when ingesting the vector.

(*ii*) *In the terrestrial environment* – the life cycle of haemogregarines comprises roughly four stages: merogony and gamogony in the vertebrate host, and fertilisation and sporogony in the invertebrate. Merogony in the vector is absent.

In addition to the classical cycle in which transmission is achieved by the bite of the vector or its ingestion by the vertebrate host, a second mode of transmission was acquired by some species: transmission by predation between vertebrates [39]. This mode of transmission is shared by all species when the alimentary diet of the host does not include the direct ingestion of the vector by the vertebrate host.

When, for example, the vector is a mosquito and the vertebrate host a snake, it is obvious that a haemogregarine cannot be transmitted regularly by ingestion of the mosquito. There must be a second vertebrate host which eats insects, develops cysts in its tissues and is part of the diet of the snake. This second host may or may not develop, in addition to cysts, the entire cycle of the parasite.

Transmission by predation is characterised for all parasites by (*i*) the absence of specificity; (*ii*) a wide repartition of infective stages (here the cyst) in the organism of the host. These principles, established for nematodes, are valid for the haemogregarines producing cysts which are disseminated in numerous organs [7].

When the vector is a mite or a tick, the sporogonic development may follow two courses: (*i*) in one step: the sporogony evolves directly from zygote to oocyst, sporoblasts and sporocysts inside the same envelop, like in *Hepatozoon perniciosum* Miller, 1908 [51] or (*ii*) in two steps: oocysts undergo the first division to produce motile sporokinetes instead of sporoblasts. Sporokinetes, after the rupture of the oocyst envelop, invade new cells of the host; sporokinetes complete their development into sporoblasts and sporocysts either in the same host, like in *Hemolivia stellata* Petit et al., 1990 [58] or in the next host generation when they invade the oocytes of the mite, like in *Karyolysus* Labbé, 1894 [35].

In both instances, the vertebrate host ingests either directly the invertebrate host or cysts from the tissues of another vertebrate host. It was suggested that sporocysts of *Hemolivia mauritanica* or *H. stellata* Petit et al., 1990 [58] might also be excreted with the faeces of the tick and be infective to susceptible hosts or transported by paratenic hosts.

As pointed out by Smith (1996) [68], a great many haemogregarines were described on the basis of gametocyte morphology and very often designated as *Hepatozoon* spp. or *Haemogregarina* spp. However, only the observation of stages in the vector may indicate the generic position of the parasite [71]. Through the years, the nomenclature has evolved, while new life cycles have been unravelled. For example, *Hemogregarina mauritanica* Sergent and Sergent, 1904 [64] studied by Laveran (1905) [41] and Brumpt (1938) [5] was renamed successively *Hepatozoon mauritanicum* by Michel (1973) [50] and later *Hemolivia mauritanica* by Landau and Paperna (1997) [40].

The genus *Hepatozoon*, well defined by the morphology and the life cycle of its type species, *H. perniciosum* Miller, 1908, has over time become a heterogeneous group of species with diverse life cycles and which, according to work by Barta et al. (2012), is paraphyletic (see Discussion) [3].

The genus *Hemolivia*, which is clearly defined by its morphological and biological features, has recently been investigated by molecular biology and its phylogenetic relationship with *Hepatozoon* studied [3, 27, 34]. A molecular analysis of two of the three *Hemolivia* species, *Hemolivia mauritanica* from *Testudo graeca* and *Testudo marginata* [27, 34], *Hemolivia mariae* from *Egernia stokesii* and *Tiliqua rugosa* [3, 34], was performed, as well as for *Hemolivia* sp. from *Rhinoclemmys Pulcherrima manni* [34]. A number of haemogregarines of reptiles and amphibians could probably be assigned to the genus *Hemolivia* if their life cycle were known.

In this communication, we (*i*) present molecular data on *H. stellata* obtained from 25-year-old archived original material; (*ii*) partially re-describe the haemogregarine *Hepatozoon argantis* Garnham, 1954 [20] and reassign it to the genus *Hemolivia*; and (*iii*) analyse the haemogregarines’ known life cycles and propose *Bartazoon* n. g.

## Materials and methods

### *Hemolivia argantis*

In 1986, PCC Garnham deposited part of his collection of slides at the Welcome Trust, including many types of haemosporidians [21] and the rest of his collection at the Muséum National d’Histoire Naturelle (MNHN) in Paris, France. In the MNHN’s collection, we found part of the original material used to describe *Hepatozoon argantis* Garnham, 1954 in the tick *Argas brumpti* Neumann, 1907 [53]. This material consists of sections of the tick with a massive infection by a haemogregarine which we identified as belonging to the genus *Hemolivia* and not *Hepatozoon*.

As we are certain that we are dealing with the original material and that only one species of haemogregarine is present, and because of the absence of type designation by the authors, this material should be considered as syntype. It consists of sections of the tick *Argas brumpti* on which the description of the haemogregarine *H. argantis* was based; the tick was identified as belonging to the original description by a photograph (Fig. 18) in Garnham 1954 [20], showing one of the sections of the tick and by drawings of oocysts of different stages. Some sections were stained by Ehrlich’s haematoxylin and eosin, some by haemalun eosin, and smears of caecal contents by Giemsa stain.

### *Hemolivia stellata*

Archived Giemsa-stained blood smears or tissue imprints have been used as a source of DNA for PCR amplification for *Plasmodium* [8, 30, 63, 79], *Leishmania* [77] and *Hepatozoon* [9] species. They provide extremely valuable material for retrospective study [30, 77] and molecular characterisation.

**Figure 1.**
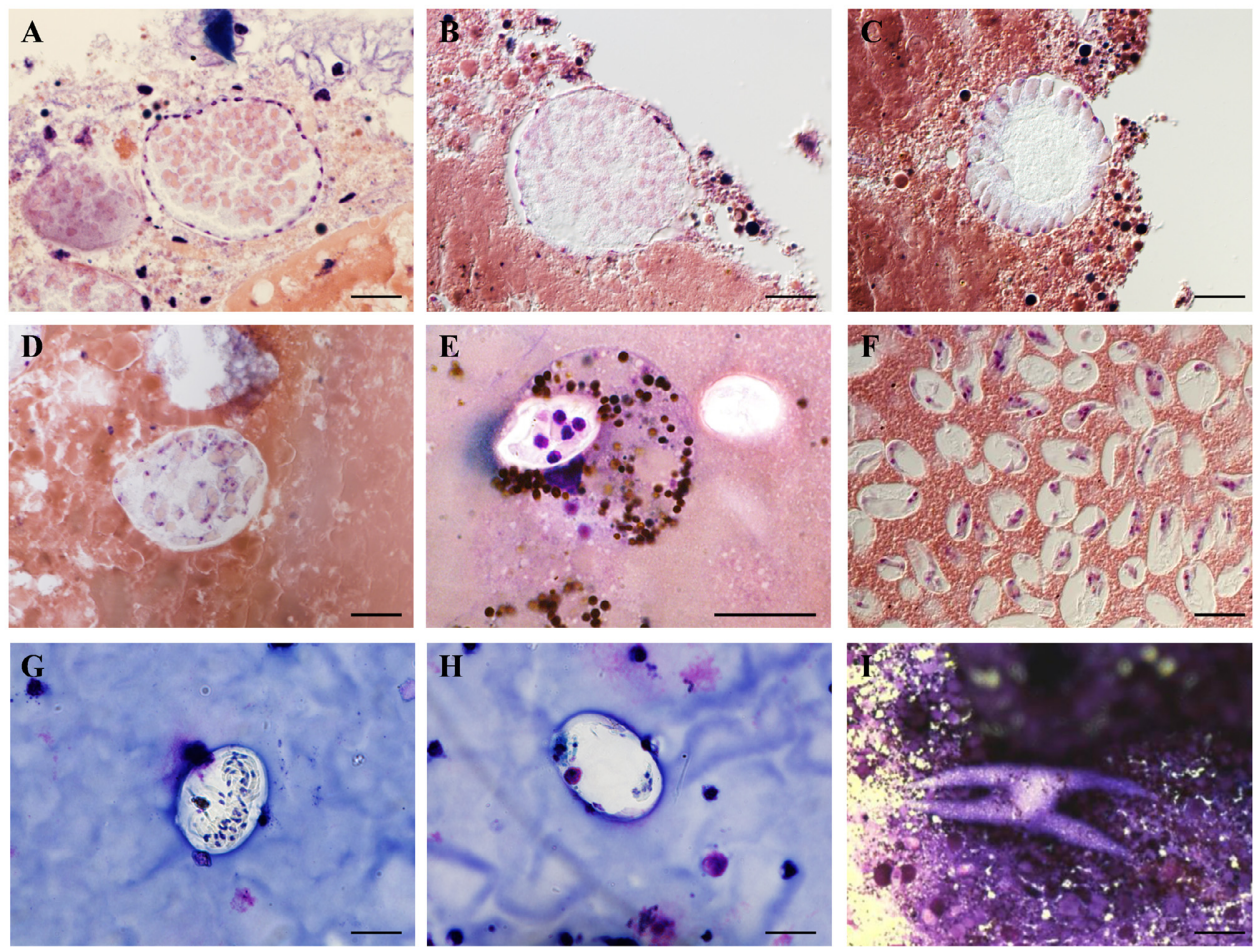
**Stages of development of *Hemolivia argantis* (A–H) and *Hemolivia stellata* (I) in their invertebrate hosts. B, C** and **F**: Nomarski. **A** and **B**: Immature oocysts with peripheral nuclei. **C**: Budding of future sporokinetes at the periphery of the oocyst. **D**: Oocyst containing sporokinetes in caecal content. **E**: Sporocyst inside a digestive cell. **F**: Mature sporocysts inside the gut contents. **G** and **H**: Sporocysts in coxal fluid. **I**: Star-shaped oocyst of *H. stellata* in the haemocoel of *Amblyomma rotondatum*. Scale bars: 20 μm.

A 25-year-old archived smear of crushed *Amblyomma rotondatum* (Koch, 1844) [31] which was part of the material used for the original description of *H. stellata*, containing many immature stellate oocysts (Fig. 1I), was used as a source of DNA for molecular characterisation of this parasite on the basis of 18S ssrRNA.

The coverslip was dismounted; the smear was scraped off the glass slide with a sterile scalpel; the material collected was incubated at 50 °C in ATL buffer containing proteinase K until total digestion; DNA was extracted using the QIAamp DNA Mini Kit from Qiagen^®^ following the manufacturer’s recommendations. DNA was eluted in 50 μL of elution buffer and frozen at −30 °C. DNA was amplified by a semi-nested PCR assay. The first amplification was performed with one pair of universal 18S rRNA oligonucleotide primers 2867 [5′-AACCTGGTTGATCCTGCCAG-3′]/2868 [5′-TGATCCTTCTGCAGGTTCACCTAC-3′], as described by Mathew et al. (2000) [47]. For the second step, two semi-nested PCRs were carried out with one external oligonucleotide primer of the first reaction paired with one internal Protozoan-specific primer derivate from Vilcins et al. [74] as follow: 2867/Hep900 [5′-CAAATCTAAGAATTTCACCTCTGAC-3′] and Hep300 [5′-GTTTCTGACCTATCAGCTTTCGACG-3′]/2868 amplifying to overlapping fragments of 939 bp and 1510 bp, respectively. The PCRs were run in a total volume of 20 μL, containing 1X High Fidelity PCR Buffer, 3 mM of MgSO_4_, 0.5 U of Platinum^®^ *Taq* DNA Polymerase High Fidelity (Invitrogen^TM^), 0.2 mM of each dNTP (Promega), 0.25 μM of each primer and 3 μL of original DNA template in the first reaction; 1 μL of the PCR product was used as a template in the second reaction. All PCRs were run on a Veriti^®^ Thermal Cycler (Applied Biosystems^®^). The PCR products were resolved by 1.5% agarose gel electrophoresis prior to purification by the QIAquick^®^ PCR Purification Kit (Qiagen^®^), following the manufacturer’s recommendations. Purified products were eluted in 30 μL of nuclease-free H_2_O and frozen at −30 °C. PCR products were prepared for sequencing in both directions, using the BigDye^®^ Terminator v3.1 Cycle Sequencing Kit (Applied Biosystems^®^) and the respective oligonucleotide primers. The BigDye^®^ reaction products were purified using the BigDye^®^ XTerminator™ Purification Kit (Applied Biosystems^®^), following the manufacturer’s recommendations before being sequenced on a 3500xl Genetic Analyzer (Applied Biosystems^®^).

Alignment and cross-checking of the sequences were performed with CLC Main Workbench 5.7 software (CLC bio) and a consensus sequence of 1816 bp was obtained by combining the two overlapping fragments. The nearly complete sequence (1816 bp) of the 18S ssrRNA gene recovered from the original archived material of *Hemolivia stellata* was deposited in GenBank under Accession Number KP881349. Our new sequence of *H. stellata* and 179 sequences of adeleid parasites (Table 2) were aligned using a Muscle algorithm [19]. Molecular phylogeny was performed by the Maximum Likelihood (ML) method with a GTR + Γ + I model, using PhyML 3.0 software [23]. Nodal robustness of the tree was evaluated by non-parametric bootstrapping (1000 replicates).

**Table 1.** Main characteristics of the four haemogregarine types and the corresponding genera.

	*Hepatozoon* Type I	*Karyolysus* Type II	*Hemolivia* Type III	*Bartazoon* n. g. Type IV
Vector	Ticks or mites	Mites	Ticks	Biting insects
Vertebrate hosts	Mammals	Reptiles	Reptiles, Amphibians	Reptiles, Amphibians, Marsupials, Birds and Rodents
Fertilisation	Syngamy	Syzygy	Syzygy	Syzygy
Sporogony	One stage	Two stages	Two stages	One stage

**Table 2.** **List of the sequences used in the phylogenetic construction.** The different columns give respectively the accession numbers of the sequences, the name of the parasites to which they are assigned, their vertebrate hosts, their isolation source, the country in which they have been found and the number of the associated reference in the references list. Unpub.: unpublished data only deposited in GenBank; a: experimentally fed on a naturally infected host; b: experimentally infected; c: collected from naturally infected wild host; d: *Amblyrhynchus cristatus* DNA detected in the last blood meal; n.a.: not available.

Accession number	Parasites	Hosts	Isolation sources	Country	References
AF130361	*Hepatozoon catesbianae*	*Lithobates catesbeianus*	n.a.	Canada	[6]
AF176835	*Hepatozoon canis*	*Canis familiaris*	*Rhipicephalus sanguineus* ^a^	India	[47]
AF176836	*Hepatozoon americanum*	*Canis familiaris*	*Amblyomma maculatum* ^a^	USA	
AF176837	*Hepatozoon catesbianae*	*Lithobates catesbeianus*	*Culex territans* ^a^	Canada	
AF297085	*Hepatozoon* sp.	*Boiga irregularis*	n.a.	Australia	Unpub.
AF494058	*Adelina bambarooniae*	*Dermolepida albohirtum*	Host larvae	Australia	Unpub.
AF494059	*Adelina bambarooniae*	*Dermolepida albohirtum*	Host larvae	Australia	
AY150067	*Hepatozoon canis*	*Vulpes vulpes*	Host spleen	Spain	[13]
AY461375	*Hepatozoon canis*	*Cerdocyon thous*	Host spleen	Brazil	
AY461376	*Hepatozoon canis*	*Lycalopex gymnocercus*	Host spleen	Brazil	
AY461377	*Hepatozoon* sp.	*Cerdocyon thous*	Host spleen	Brazil	
AY461378	*Hepatozoon canis*	*Canis familiaris*	Host blood	Spain	
AY471615	*Hepatozoon* sp.	*Lycalopex gymnocercus*	Host spleen	Brazil	
AY600625	*Hepatozoon* cf. *erhardovae*	*Clethrionomys glareolus*	Host blood	Spain	
AY600626	*Hepatozoon* cf. *erhardovae*	*Clethrionomys glareolus*	Host blood	Spain	
AY620232	*Hepatozoon felis*	*Felis catus*	Host blood	Spain	
AY628681	*Hepatozoon felis*	*Felis catus*	Host blood	Spain	
AY731062	*Hepatozoon canis*	*Vulpes vulpes*	Host spleen	Spain	
DQ096835	*Adelina dimidiata*	*Scolopendra cingulata*	Host faeces	Bulgaria	[32]
DQ096836	*Adelina grylli*	*Gryllus bimaculatus*	Host fat body^b^		
DQ111754	*Hepatozoon canis*	*Canis familiaris*	Host blood	Sudan	[55]
DQ439540	*Hepatozoon canis*	*Canis familiaris*	Host blood	Venezuela	[12]
DQ439541	*Hepatozoon canis*	*Vulpes vulpes*	Host spleen	Spain	
DQ439543	*Hepatozoon canis*	*Canis familiaris*	Host blood	Venezuela	
EF125058	Reported to be host’s DNA	*Cerastes cerastes*	n.a.	Saudi Arabia	Unpub.
EF157822	*Hepatozoon ayorgbor*	*Python regius*	*Culex quinquefasciatus* ^a^	Ghana	[66]
EF222257	*Hepatozoon* sp.	*Martes martes*	Host blood	Spain	[11]
EF222259	*Hepatozoon* sp.	*Sciurus vulgaris*	Host blood	Spain	
EU041717	*Hepatozoon ursi*	*Ursus thibetanus japonicus*	Host lung and blood	Japan	[33]
EU041718	*Hepatozoon ursi*	*Ursus thibetanus japonicus*	Host lung	Japan	
EU289222	*Hepatozoon canis*	*Canis familiaris*	n.a.	Taiwan	Unpub.
EU430231	*Hepatozoon* sp.	*Varanus panoptes*	*Amblyomma fimbriatum* ^c^	Australia	[76]
EU430232	*Hepatozoon* sp.	*Varanus panoptes*	*Amblyomma fimbriatum* ^c^	Australia	
EU430233	*Hepatozoon* sp.	*Liasis fuscus*	*Amblyomma moreliae* ^c^	Australia	
EU430234	*Hepatozoon* sp.	*Varanus panoptes*	*Amblyomma fimbriatum* ^c^	Australia	
EU430235	*Hepatozoon* sp.	*Varanus panoptes*	*Amblyomma fimbriatum* ^c^	Australia	
EU430236	*Hepatozoon* sp.	*Liasis fuscus*	*Amblyomma fimbriatum* ^c^	Australia	
EU430237	*Hepatozoon* sp.	*Sarcophilus harrisii*	*Ixodes tasmani* ^c^	Australia	[75]
EU430238	*Hepatozoon* sp.	*Sarcophilus harrisii*	*Ixodes tasmani* ^c^	Australia	
FJ719813	*Hepatozoon* sp.	*Dromiciops gliroides*	Host blood	Chile	[49]
FJ719814	*Hepatozoon* sp.	*Dromiciops gliroides*	Host blood	Chile	
FJ719815	*Hepatozoon* sp.	*Abrothrix olivaceus*	Host blood	Chile	
FJ719816	*Hepatozoon* sp.	*Abrothrix sanborni*	Host blood	Chile	
FJ719817	*Hepatozoon* sp.	*Abrothrix olivaceus*	Host blood	Chile	
FJ719818	*Hepatozoon* sp.	*Abrothrix olivaceus*	Host blood	Chile	
FJ719819	*Hepatozoon* sp.	*Abrothrix sanborni*	Host blood	Chile	
HM212625	*Hepatozoon canis*	*Vulpes vulpes*	Host spleen	Croatia	[17]
HM212626	*Hepatozoon canis*	*Vulpes vulpes*	Host spleen	Croatia	
HQ224954	*Hepatozoon* cf. *catesbianae*	*Lithobates catesbeianus*	Host blood	Canada	[3]
HQ224955	*Klossia helicina*	*Cepaea nemoralis*	Host tissue	France	
HQ224956	*Klossia helicina*	*Cepaea nemoralis*	Host tissue	France	
HQ224957	*Dactylosoma ranarum*	*Pelophylax* kl. *esculentus*	Host blood	France	
HQ224958	*Dactylosoma ranarum*	*Pelophylax* kl. *esculentus*	Host blood	France	
HQ224959	*Haemogregarina balli*	*Chelydra serpentina*	Host blood	Canada	
HQ224960	*Hepatozoon magna*	*Pelophylax* kl. *esculentus*	Host blood	France	
HQ224961	*Babesiosoma stableri*	*Lithobates septentrionalis*	Host blood	Canada	
HQ224962	*Hepatozoon* cf. *clamatae*	*Lithobates clamitans*	Host blood	Canada	
HQ224963	*Hepatozoon* cf. *clamatae*	*Lithobates clamitans*	Host blood	Canada	
HQ292771	*Hepatozoon* sp.	*Tachylepis wrightii*	Host tail tissue and blood	Seychelles	[28]
HQ292772	*Hepatozoon* sp.	*Tachylepis wrightii*	Host tail tissue and blood	Seychelles	
HQ292773	*Hepatozoon* sp.	*Lycognathophis seychellensis*	Host tail tissue and blood	Seychelles	
HQ292774	*Hepatozoon* sp.	*Lycognathophis seychellensis*	Host tail tissue and blood	Seychelles	
HQ292775	*Hepatozoon* sp.	*Lycognathophis seychellensis*	Host tail tissue and blood	Seychelles	
HQ734787	*Hepatozoon* sp.	*Tarentola mauritanica*	Host tail tissue with blood	Algeria	[45]
HQ734788	*Hepatozoon* sp.	*Tarentola mauritanica*	Host tail tissue with blood	Algeria	
HQ734789	*Hepatozoon* sp.	*Quedenfeldtia moerens*	Host tail tissue with blood	Morocco	
HQ734790	*Hepatozoon* sp.	*Ptyodactylus oudrii*	Host tail tissue with blood	Algeria	
HQ734791	*Hepatozoon* sp.	*Scelarcis perspicillata*	Host tail tissue with blood	Morocco	
HQ734792	*Hepatozoon* sp.	*Podarcis vaucheri*	Host tail tissue with blood	Morocco	
HQ734793	*Hepatozoon* sp.	*Podarcis vaucheri*	Host tail tissue with blood	Morocco	
HQ734794	*Hepatozoon* sp.	*Podarcis vaucheri*	Host tail tissue with blood	Morocco	
HQ734795	*Hepatozoon* sp.	*Podarcis vaucheri*	Host tail tissue with blood	Morocco	
HQ734796	*Hepatozoon* sp.	*Eumeces algeriensis*	Host tail tissue with blood	Morocco	
HQ734797	*Hepatozoon* sp.	*Eumeces algeriensis*	Host tail tissue with blood	Morocco	
HQ734798	*Hepatozoon* sp.	*Atlantolacerta andreanskyi*	Host tail tissue with blood	Morocco	
HQ734799	*Hepatozoon* sp.	*Timon tangitanus*	Host tail tissue with blood	Morocco	
HQ734800	*Hepatozoon* sp.	*Timon tangitanus*	Host tail tissue with blood	Morocco	
HQ734801	*Hepatozoon* sp.	*Timon tangitanus*	Host tail tissue with blood	Morocco	
HQ734802	*Hepatozoon* sp.	*Timon tangitanus*	Host tail tissue with blood	Morocco	
HQ734803	*Hepatozoon* sp.	*Podarcis vaucheri*	Host tail tissue with blood	Morocco	
HQ734804	*Hepatozoon* sp.	*Podarcis vaucheri*	Host tail tissue with blood	Morocco	
HQ734805	*Hepatozoon* sp.	*Chalcides polylepis*	Host tail tissue with blood	Morocco	
HQ734806	*Hepatozoon* sp.	*Tarentola mauritanica*	Host tail tissue with blood	Morocco	
HQ734807	*Hepatozoon* sp.	*Timon tangitanus*	Host tail tissue with blood	Morocco	
HQ734808	*Hepatozoon* sp.	*Ptyodactylus oudrii*	Host tail tissue with blood	Morocco	
HQ734809	*Hepatozoon* sp.	*Quedenfeldtia moerens*	Host tail tissue with blood	Morocco	
HQ829430	*Hepatozoon ursi*	*Melursus ursinus*	Host blood	India	[56]
HQ829432	*Hepatozoon ursi*	*Melursus ursinus*	Host blood	India	
HQ829434	*Hepatozoon ursi*	*Melursus ursinus*	Host blood	India	
HQ829436	*Hepatozoon ursi*	*Melursus ursinus*	Host blood	India	
HQ829445	*Hepatozoon felis*	*Panthera tigris*	Host blood	India	[57]
HQ829446	*Hepatozoon felis*	*Panthera tigris*	Host blood	India	
HQ829447	*Hepatozoon canis*	*Cuon alpinus*	Host blood	India	
HQ829448	*Hepatozoon canis*	*Cuon alpinus*	Host blood	India	
JN181157	*Hepatozoon sipedon*	*Rana* spp. *& Nerodia sipedon*	Host blood	Canada	[3]
JN584475	*Hepatozoon felis*	*Felis catus*	Host blood	India	[57]
JN584476	*Hepatozoon felis*	*Felis catus*	Host blood	India	
JQ080302	*Hepatozoon* sp.	not confirmed^d^	*Aedes taeniorhynchus*	Ecuador	[4]
JQ080303	*Hepatozoon* sp.	not confirmed^d^	*Aedes taeniorhynchus*	Ecuador	
JQ080304	*Hepatozoon* sp.	unkown	*Aedes taeniorhynchus*	Ecuador	
JQ746622	*Hepatozoon garnhami*	*Psammophis schokari*	Host blood	Saudi Arabia	[1]
JX244266	*Hepatozoon* sp.	*Malpolon monspessulanus*	Host tail muscle tissue	Morocco	[72]
JX244267	*Hepatozoon* sp.	*Hemorrhois hippocrepis*	Host tail muscle tissue	Spain	
JX244268	*Hepatozoon* sp.	*Hemorrhois hippocrepis*	Host tail muscle tissue	Morocco	
JX244269	*Hepatozoon* sp.	*Hemorrhois hippocrepis*	Host tail muscle tissue	Morocco	
JX531910	*Hepatozoon* sp.	*Podarcis hispanicus*	Host tail tissue with blood	Spain	[46]
JX531917	*Hepatozoon* sp.	*Podarcis hispanicus*	Host tail tissue with blood	Spain	
JX531920	*Hepatozoon* sp.	*Podarcis lilfordi*	Host tail tissue with blood	Spain	
JX531921	*Hepatozoon* sp.	*Podarcis bocagei*	Host tail tissue with blood	Portugal	
JX531928	*Hepatozoon* sp.	*Podarcis bocagei*	Host tail tissue with blood	Portugal	
JX531930	*Hepatozoon* sp.	*Podarcis bocagei*	Host tail tissue with blood	Portugal	
JX531931	*Hepatozoon* sp.	*Podarcis bocagei*	Host tail tissue with blood	Portugal	
JX531932	*Hepatozoon* sp.	*Podarcis bocagei*	Host tail tissue with blood	Portugal	
JX531933	*Hepatozoon* sp.	*Algyroides marchi*	Host tail tissue with blood	Spain	
JX531940	*Hepatozoon* sp.	*Algyroides marchi*	Host tail tissue with blood	Spain	
JX531941	*Hepatozoon* sp.	*Algyroides marchi*	Host tail tissue with blood	Spain	
JX531953	*Hepatozoon* sp.	*Podarcis bocagei*	Host tail tissue with blood	Spain	
KC342524	*Hepatozoon cuestensi*	*Crotalus durissus terrificus*	Host blood	Brazil	[54]
KC342525	*Hepatozoon massardii*	*Crotalus durissus terrificus*	Host blood	Brazil	
KC342526	*Hepatozoon cevapii*	*Crotalus durissus terrificus*	Host blood	Brazil	
KC342527	*Hepatozoon cuestensi*	*Crotalus durissus terrificus*	Host blood	Brazil	
KC342528	*Hepatozoon cuestensi*	*Crotalus durissus terrificus*	Host blood	Brazil	
KC512766	*Hemolivia* sp. [*mauritanica*]	*Testudo graeca*	*Hyalomma aegyptium* ^c^	Algeria	[27]
KC696564	*Hepatozoon* sp.	*Psammophis schokari*	Host tail muscle tissue	Morocco	[73]
KC696565	*Hepatozoon* sp.	*Psammophis schokari*	Host tail muscle tissue	Morocco	
KC696566	*Hepatozoon* sp.	*Psammophis aegyptius*	Host tail muscle tissue	Niger	
KC696567	*Hepatozoon* sp.	*Psammophis sibilans*	Host tail muscle tissue	Burkina Faso	
KC696568	*Hepatozoon* sp.	*Psammophis elegans*	Host tail muscle tissue	Mali	
KC696569	*Hepatozoon* sp.	*Psammophis schokari*	Host tail muscle tissue	Algeria	
KC848055	*Hepatozoon* sp.	*Hipposideros cervinus*	Host liver	Malaysia	[59]
KC848056	*Hepatozoon* sp.	*Hipposideros cervinus*	Host liver	Malaysia	
KC848057	*Hepatozoon* sp.	*Hipposideros cervinus*	Host liver	Malaysia	
KF022102	*Hepatozoon peircei*	*Hydrobates melania*	Host blood	Mexico	[48]
KF246565	*Hepatozoon seychellensis*	*Grandisonia alternans*	Host blood	Seychelles	[26]
KF246566	*Hepatozoon seychellensis*	*Grandisonia alternans*	Host blood	Seychelles	
KF257924	*Haemogregarina* sp.	*Pelusios marani*	Host blood	Gabon	[18]
KF257926	*Haemogregarina stepanowi*	*Mauremys caspica*	Host blood	Iran	
KF939620	*Hepatozoon chinensis*	*Elaphe carinata*	Host blood	China	[24]
KF992697	*Haemogregarina stepanowi*	*Mauremys caspica*	Host blood	Turkey	[34]
KF992698	*Hemolivia mauritanica*	*Testudo graeca*	Host blood	Turkey	
KF992699	*Hemolivia mauritanica*	*Testudo marginata*	Host blood	Greece	
KF992700	*Hemolivia mauritanica*	*Testudo graeca*	Host blood	Iraq	
KF992701	*Hemolivia mauritanica*	*Testudo graeca*	Host blood	Syria	
KF992702	*Hemolivia mauritanica*	*Testudo graeca*	Host blood	Syria	
KF992703	*Hemolivia mauritanica*	*Testudo graeca*	Host blood	Syria	
KF992704	*Hemolivia mauritanica*	*Testudo graeca*	Host blood	Syria	
KF992705	*Hemolivia mauritanica*	*Testudo graeca*	Host blood	Syria	
KF992706	*Hemolivia mauritanica*	*Testudo graeca*	Host blood	Syria	
KF992707	*Hemolivia mauritanica*	*Testudo graeca*	Host blood	Syria	
KF992708	*Hemolivia mauritanica*	*Testudo graeca*	Host blood	Syria	
KF992709	*Hemolivia mauritanica*	*Testudo graeca*	Host blood	Syria	
KF992710	*Hemolivia mauritanica*	*Testudo marginata*	Host blood	Greece	
KF992711	*Hemolivia mariae*	*Egernia stokesii*	Host blood	Australia	
KF992712	*Hemolivia mariae*	*Egernia stokesii*	Host blood	Australia	
KF992713	*Hemolivia* sp. [*pulcherrima*]	*Rhinoclemmys pulcherrima*	Host blood	Nicaragua	
KF992714	*Hemolivia* sp. [*pulcherrima*]	*Rhinoclemmys pulcherrima*	Host blood	Nicaragua	
KJ189390	*Hepatozoon* sp.	*Podarcis bocagei* & *hispanica*	Host blood	Portugal	[44]
KJ189418	*Hepatozoon* sp.	*Podarcis bocagei* & *hispanica*	Host blood	Portugal	
KJ189426	*Hepatozoon* sp.	*Podarcis bocagei* & *hispanica*	Host blood	Portugal	
KJ461939	*Karyolysus* sp.	*Podarcis muralis*	Host blood	Slovakia	[24]
KJ461940	*Karyolysus* sp.	*Lacerta agilis*	Host blood	Poland	
KJ461941	*Karyolysus* sp.	*Lacerta viridis*	*Ixodes ricinus* ^c^	Hungary	
KJ461942	*Karyolysus* sp.	*Lacerta trilineata*	Host blood	Romania	
KJ461943	*Karyolysus* sp.	*Lacerta viridis*	Host blood	Hungary	
KJ461944	*Karyolysus* sp.	*Lacerta viridis*	*Ophionyssus* sp. ^c^	Hungary	
KJ461945	*Karyolysus* sp.	*Zootoca vivipara*	*Ophionyssus* sp. ^c^	Poland	
KJ461946	*Karyolysus* sp.	*Zootoca vivipara*	Host blood	Poland	
KJ702453	*Hepatozoon fitzsimonsi*	*Chersina angulata*	Host blood	South Africa	[9]
KM234646	*Hepatozoon domerguei*	*Madagascarophis colubrinus*	Host tail muscle tissue	Madagascar	[43]
KM234647	*Hepatozoon domerguei*	*Madagascarophis colubrinus*	Host tail muscle tissue	Madagascar	
KM234648	*Hepatozoon domerguei*	*Ithycyphus oursi*	Host tail muscle tissue	Madagascar	
KM234649	*Hepatozoon domerguei*	*Furcifer* sp.	Host tail muscle tissue	Madagascar	
KP119770	*Hepatozoon ixoxo*	*Amietophrynus garmani*	Host blood	South Africa	[52]
KP119771	*Hepatozoon ixoxo*	*Amietophrynus gutturalis*	Host blood	South Africa	
KP119772	*Hepatozoon ixoxo*	*Amietophrynus maculatus*	Host blood	South Africa	
KP119773	*Hepatozoon theileri*	*Amietia quecketti*	Host blood	South Africa	
KR069082	*Hemolivia parvula*	*Kinixys zombensis*	Host blood	South Africa	[10]
KR069083	*Hemolivia parvula*	*Kinixys zombensis*	Host blood	South Africa	
KR069084	*Hepatozoon fitzsimonsi*	*Kinixys zombensis*	Host blood	South Africa	

## Results

### Life cycles and morphology

*H. argantis* was described in detail by Garnham, 1954 [20] and we will only provide a general outline: (*i*) in the haemocoelom: large immature oocysts with peripheral nuclei (Figs. 1A & 1B); (*ii*) round, immature oocysts with sporokinetes budding at the periphery of a large cytoplasmic mass (Fig. 1C) or, apparently free in the oocyst (Fig. 1D); and (*iii*) sporocysts filled with sporozoites in the haemocoelom (Fig. 1F) and the gut contents of the tick.

In the re-examined material, we also found a few intra-cellular sporocysts beneath the intestinal epithelium and inside desquamated cells of the gut (Fig. 1E), suggesting that the initial site of development of sporokinetes into sporocysts is intra-cellular.

*Hemolivia stellata* was described by Petit et al., 1990 [58]. Schizonts of this species occur in erythrocytes and leucocytes of the cane toad *Rhinella marina* (Linnaeus, 1758) (as *Bufo marinus*) and gametocytes of this species occur in erythrocytes of this amphibian. Oocysts in the tick, *Amblyomma rotondatum*, are star shaped, and release sporokinetes that migrate to new intestinal cells and mature into sporocysts; many sporocysts are found in the intestinal fluid.

### Parasite sequences and phylogenetic analysis

The phylogenetic analysis included 166 published sequences of *Hepatozoon* (140), *Karyolysus* (8) and *Hemolivia* (18) parasites isolated from a variety of vertebrates, 13 other published sequences of Adeleidae as outgroups to root the tree and our sequence of *H. stellata* (Table 2).

**Figure 2.**
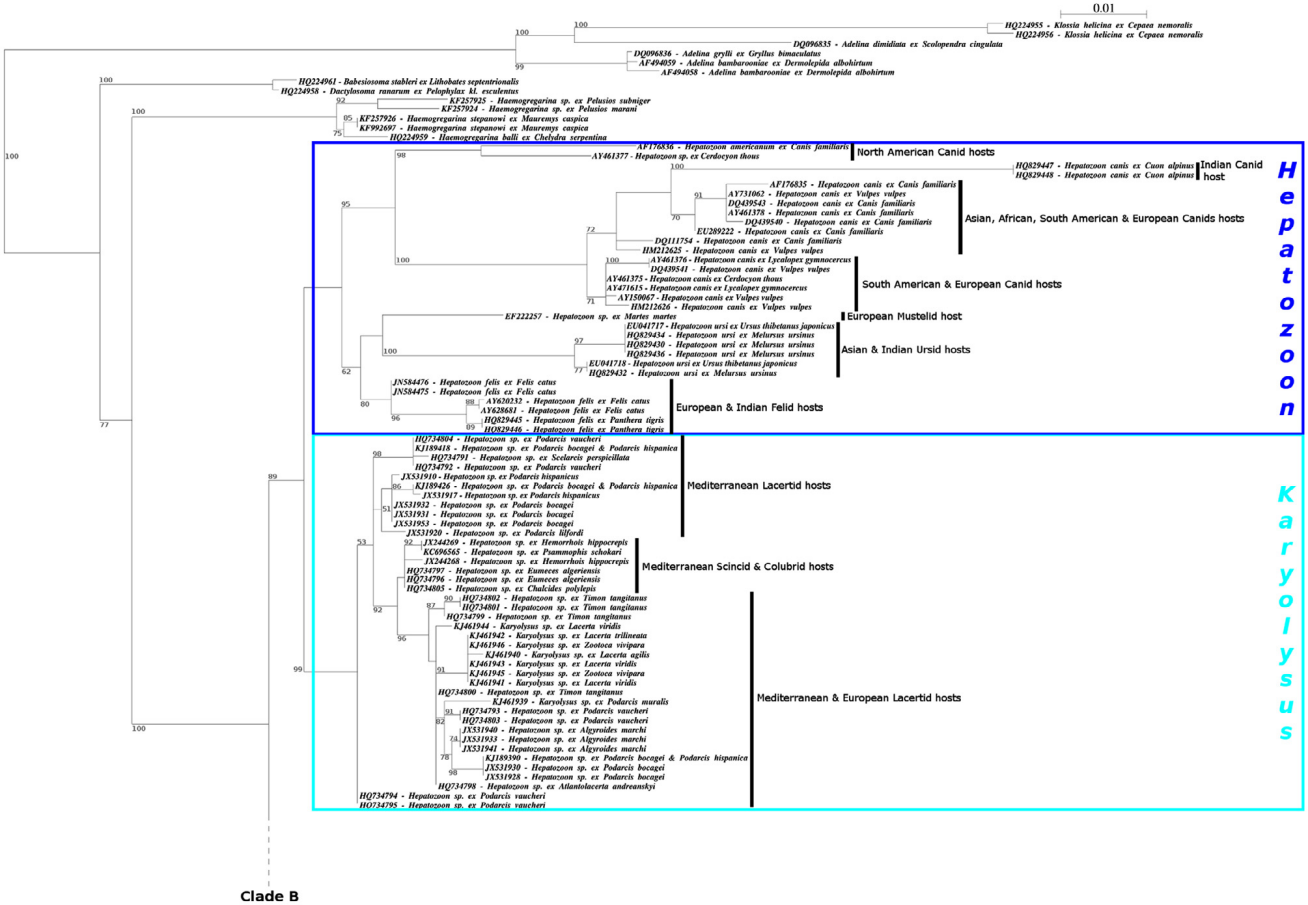
**Phylogenetic tree based on the 18S ssrDNA gene of adeleorinid Coccidia illustrating the polyphyly of the genus *Hepatozoon* and the new proposed classification of the terrestrial Haemogregarines.** Analysis, generated by the Maximum Likelihood (ML) method with a GTR + Γ + I model, performed with 180 sequences: 6 monoxenous parasites (4 *Adelina* and 2 *Klossia*) as outgroup and 173 heteroxenous parasites (139 *Hepatozoon*, 18 *Hemolivia*, 8 *Karyolysus*, 5 *Haemogregarina*, 1 *Dactylosoma*, 1 *Babesiosoma*, 1 sequence extracted from the host *Cerastes cerastes*), all downloaded from GenBank, and our new sequence of *Hemolivia stellata*. The intermediate host is indicated for each sequence. The family of the Vertebrate hosts and the geographical origin are in boldface characters. Coloured boxes indicate the type/genus of the terrestrial haemogregarines: Haemogregarines of Type I – genus *Hepatozoon* in dark blue; Haemogregarines of Type II – genus *Karyolysus* in light blue; Haemogregarines of Type III – genus *Hemolivia* in red; Haemogregarines of Type IV – genus *Bartazoon* in green. Nodal support is provided by bootstrap values, estimated by 1000 replicates and only shown when > 50%. Hypothesised evolutionary changes can be evaluated with the scale bar.

A robust *Hemolivia* clade was obtained with *H. stellata* at the base (Fig. 2). It comprises all the *Hemolivia* sequences included in the phylogenetic construction but also a few sequences from *Hepatozoon* isolated in Australia from *Varanus panoptes* (Storr, 1980) and from *Liasis fuscus* Peters, 1873.

What was previously considered as the *Hepatozoon* group was paraphyletic. It was clearly divided into two major clades (Fig. 2).

The first clade was subdivided into two robust subclades containing respectively the *Hepatozoon* from carnivores and a mixed group containing some of the *Hepatozoon* of Mediterranean reptiles and the *Karyolysus* of the European lacertids.

The second clade was also subdivided, with the *Hemolivia* clade on the one hand and a group containing sequences of haemogregarines of reptiles and amphibians transmitted by biting insects (Diptera and Siphonaptera) on the other.

A sequence of a haemogregarine from the bird *Oceanodroma melania* (Bonaparte, 1854) was found at the base of this group, as well as a monophyletic group of sequences of parasites from Australian and South American marsupials, a monophyletic group of sequences from amphibian hosts from various geographic areas, a few sequences of haemogregarines from rodents and three sequences of parasites extracted from the liver of the bats *Hipposideros cervinus* (Gould, 1863) [59].

### Taxonomic summaries

*Hemolivia argantis* (Garnham, 1954) n. comb.

(=*Hepatozoon argantis* Garnham, 1954).

Host: *Argas brumpti.*

Locality: Egypt.

Syntype: Sections of a tick and smears of caecal contents. Collection number: MNHN PIV 169-173, 179-182, 184-200 (251YY).

### *Bartazoon* n. gen.

urn:lsid:zoobank.org:act:55D637B3-A9D9-4C01-92A4-E0B4F0052958

Type species: *Bartazoon breinli* (Mackerras, 1960) n. comb. (=*Hepatozoon breinli* Mackerras, 1960).

Type host of the type species: *Varanus tristis orientalis.*

Type locality of the type species: Innisfail, Queensland, Australia.

Etymology: named after John R. Barta (University of Guelph, Canada), in recognition of his contribution to the biology of haemogregarines.

Definition: Haemogregarines of Type 4: infecting various vertebrate hosts, vectorised by biting insects, fertilisation by syzygy.

## Discussion

### Taxonomic status of *H. argantis*

It appears clearly that the parasite in *Argas* does not belong to the genus *Hepatozoon* in which the sporoblasts mature into sporocysts inside the oocyst envelop. The sporogony evolves in two stages: (i) mature oocysts release motile sporokinetes, and (ii) sporokinetes invade the cells of the tick and develop into sporoblasts and sporocysts.

Garnham in 1954 [20] did point to differences with the classical cycle of *Hepatozoon* and compared the parasite from *Argas* with another genus existing at the time, *Karyolysus* (Danilewskyi, 1886) [15], which also produces sporokinetes in a mite *Lyponyssus*. However, in the latter, sporokinetes invade the oocytes of the mite and mature only in the next generation. The authors choose to assign the haemogregarine in *Argas* to the genus *Hepatozoon* but noted that it might be a new genus. It is now clear that it belongs to the genus *Hemolivia*.

### Host spectrum

The vectors, definitive hosts of terrestrial haemogregarines, fall into two groups: insects (Diptera and fleas) on the one hand, and haematophagous Acari (ticks and mites) on the other.

Mosquitoes and fleas are considered as hosts for *Hepatozoon* while Acari are vectors of the three existing genera: *Hepatozoon*, *Karyolysus* and *Hemolivia*.

*Hepatozoon* is at present a large gathering of species classified in this genus on the sole basis of the presence of gametocytes in the blood. Some species, previously considered as *Hepatozoon*, when their life cycles were unravelled, were assigned to the genus *Hemolivia*: *H. mauritanica*, *H. mariae*, *H. stellata* and here *H. argantis.* The development in the vector of many haemogregarines follows different courses which will be discussed in the next section.

### Life cycles

The fundamental life cycle of a Coccidiomorpha consists of (i) the infective stage, i.e. the sporozoite, (ii) male and female gametes, and (iii) the zygote. Many adaptive additions to this simple scheme arose either to multiply the parasite in the vertebrate host (schizogony) or in the vector (sporogony) or to facilitate transmission according to the hosts and their life habits: free resistant stages ingested in the external environment, infective stages ingested by a paratenic host, bite by a vector and predation between vertebrate hosts.

The adeleids’ fertilisation procedures, including syzygy, gametogenesis with maturation of a macrogamete and production of a small number of microgametes inside a common envelope, followed by the fertilisation of the macrogamete, were reported from several haemogregarines considered as *Hepatozoon* and *Hemolivia*.

Another mode of fertilisation is described in *Hepatozoon* of mammals transmitted by ticks or mites: *Hepatozoon perniciosum* Miller, 1908 [51] of the rat, the type species of the genus, *H. canis*, and *H. americanum* from Canidae: syngamy, which is the association of a pair of male and female gametes and their fusion without production of flagellate microgametes.

In the literature, syngamy was described in the gregarine *Coelogregarina ephestiae* Ghelelovitch, 1948 [22] from *Ephestia kuehniella* (Zeller, 1879). It would be a unique example of syngamy in the Gregarines and the question of their classification within the Gregarinomorpha or the Coccidiomorpha may be considered.

### Molecular data of *Hemolivia*

The molecular data deposited under the name *Hemolivia* into the databanks reaches 21 sequences of the 18S ribosomal RNA gene:

(*i*) The sequence JN211118 deposited by Barta et al. (2012) [3] as *Hemolivia mariae* Smallridge & Paperna, 1997 [67] was isolated from dried blood films containing gamonts from an experimentally infected *Tiliqua rugosa* Gray, 1825 from Australia. This sequence is not included into our tree (Fig. 2) because of its shortness and of poor overlap with the rest of the sequences. However, it clusters with the *Hemolivia* sequences that are long enough to overlap (data not shown). In Barta et al. (2012) [3], it also clusters with the sequence “*Hepatozoon*” sp. EU430236 that belongs to the *Hemolivia* clade in Kvičerová et al. (2014) [34] and in our analyses (Fig. 2).

(*ii*) The sequence HQ224961 also deposited by Barta et al. (2012) [3] as *Hemolivia mariae* Smallridge and Paperna, 1997 [67] is mistakenly referenced in GenBank while it is clearly stated in the text of the article that it is a sequence of *Babesiosoma stableri* Schmittner & McGhee, 1961 [62] obtained from *Rana septentrionalis* Baird, 1854 collected by hand from Lake Sasajewun, Algonquin Provincial Park, Ontario, Canada. As reported [3, 34], this sequence also clusters in our analysis (Fig. 2) with the related *Dactylosoma ranarum* Labbé, 1894 [35].

(*iii*) The sequence KC512766 deposited as *Hemolivia* sp. by Harris et al. (2013) [27] was isolated from *Hyalomma aegyptium* Linnaeus, 1758 collected on *Testudo graeca* Linnaeus, 1758 in Algeria, the original host and locality of *H. mauritanica.*

(*iv*) The sequence KF270674 deposited as *Hemolivia* sp. by William et al. (2014) [78] was isolated from the blood of *Panthera leo* (Linnaeus, 1758) from Zambia. This sequence is not included in our tree (Fig. 2) because of its shortness (298 bp) and in addition appears more related to sequences of *Adelina* and *Dactylosoma* than to sequences of *Hemolivia* by BLAST [2].

(*v*) The 13 sequences KF992698 – KF992710 deposited as *Hemolivia mauritanica* by Kvičerová et al. (2014) [34] were isolated from *Hyalomma aegyptium* L., 1758 collected on *T. graeca* from Algeria, Iraq, Syria and Turkey, and on *Testudo marginata* Shoepf, 1789 from Greece. All these sequences cluster with the sequence KC512766 (Fig. 2) and seem to correspond to *Hemolivia mauritanica* and some of its variants.

(*vi*) The two sequences KF992711 – KF992712 deposited as *Hemolivia mariae* by Kvičerová et al. (2014) [34] were isolated from *Amblyomma* sp. and *Bothriocroton* sp. collected on *Egernia stokesii* (Gray, 1845) from South Australia. They were collected from the original location and the same vertebrate host but in a different vector. As reported by Kvičerová et al. (2014) [34], these two sequences cluster with some of the *Hepatozoon* sp. from the Australian Reptiles.

(*vii*) The two sequences KF992713 – KF992714 deposited as *Hemolivia* sp. by Kvičerová et al. (2014) [34] were isolated from the blood of *Rhinoclemmys pulcherrima manni* (Dunn, 1930) from Nicaragua. These sequences cluster with the *Hemolivia mauritanica* clade.

(*viii*) The two sequences KR069082 – KR069083 deposited as *Hemolivia parvula* by Cook *et al.* 2015 were isolated from the blood of *Kinixys zombensis* Hewit, 1931 from South Africa and as shown by the authors and in our analyses, belong to the *Hemolivia* clade and cluster with the *Hemolivia* of Mediterranean turtles.

Finally, *Karyolysus* and *Hemolivia* have a similar life cycle in two stages but they differ in several respects, such as the vector (according to present knowledge): mite vs. tick; the vertebrate host: lizard vs. turtle; trans-ovarian transmission vs. direct transmission. Molecular biology shows the two genera to be separated into two distinct clades with a well bootstrap value for clade A (in which the sequences of *Karyolysus* are found) but not for clade B (in which the sequences of *Hemolivia* are found).

### Phylogenetic analyses

Our analyses show the phylogenetic position of *H. stellata* to be at the base of a robust *Hemolivia* clade. This clade supports the monophyly of the genus *Hemolivia*, as previously reported [3, 34], and includes several parasites assigned to the genus *Hepatozoon*: EU430231, EU430232 isolated from *Varanus panoptes* (Storr, 1980), EU430236 isolated from *Liasis fuscus* Peters, 1873 from Australia [76]. When more information on their life cycle is known, they might be reassigned to *Hemolivia*.

Siddall (1995) [65], Smith and Desser (1997) [69], analysing the morphology and life cycle traits of *Hepatozoon* species, thought that they consist of different genera. Barta et al. (2012), Kvičerová et al. (2014), using molecular tools came to the same conclusion [3, 34]. Analyses also agree on the paraphyly of the genus *Hepatozoon* that contains the *Hemolivia* clade and appears clearly divided into two major clusters (Fig. 2). The first one is subdivided into two robust clades containing respectively the *Hepatozoon* from carnivores (=Clade A in Kvičerová et al. (2014) [34]) and some of the *Hepatozoon* of Mediterranean reptiles essentially lacertids, colubrids and scincids (=Clade B in Kvičerová et al. (2014) [34]) in mix with the *Karyolysus* of the European lacertids (Haklová-Kočíková et al. 2014). Three other sequences of *Karyolysus* are found in Clade B. They were sequenced from ticks but there is no evidence of a complete cycle in the tick; so they may belong neither to the *Hepatozoon* genus (in which they are assigned) nor to the *Karyolysus*. The second one is also dual with the *Hemolivia* clade on the one hand and the *Bartazoon* (=Clade C in Kvičerová et al. (2014) [34]) on the other. This latter clade contains several clusters: (*i*) parasites from bird hosts; (*ii*) parasites of marsupials grouping hosts from Australia and South America; (*iii*) some parasites from the South American viperid; (*iv*) some parasites of Mediterranean reptiles mixed with the rest of the parasites from the South American Viperid; (*v*) the parasites of the ranids, grouping hosts from North America and Europe; and (*vi*) the parasites from Madagascan caecillid hosts; the rest forms a broad-range-host cluster containing parasites from diverse geographic areas, essentially isolated from reptiles and rodents, and from bats.

**Figure 3.**
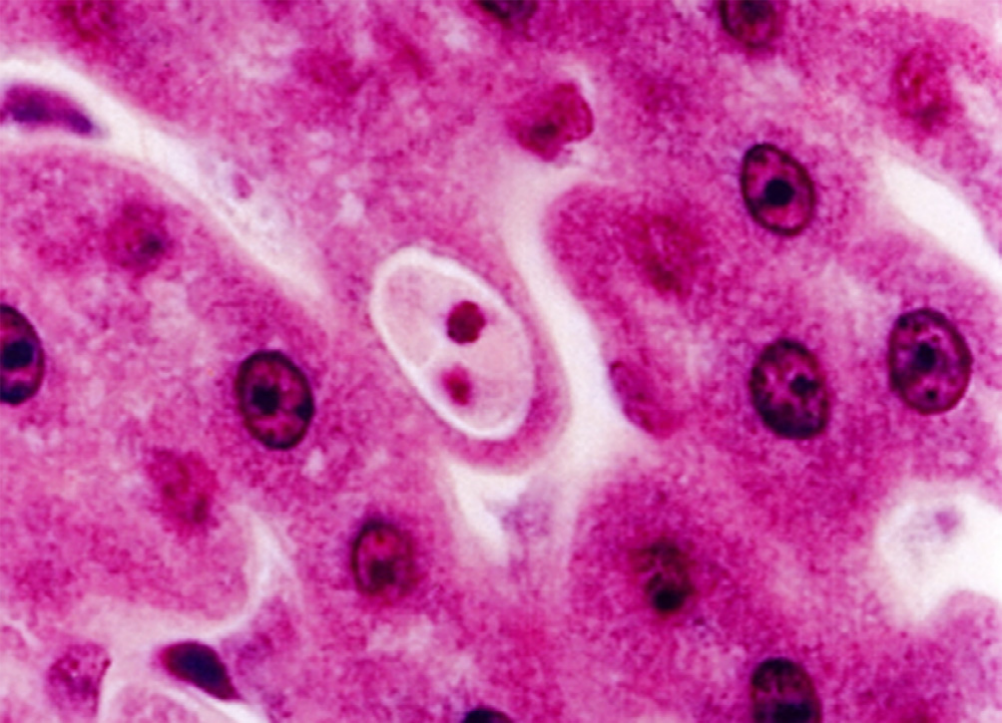
Cyst of a haemogregarine in the liver of a *Miniopterus inflatus* from Gabon.

The nature of the haemogregarine described in the lizard *Sphenodon punctatus* and the associated tick *Amblyomma sphenodonti* in New Zealand remains unsolved. The lizard is considered as the only surviving member of the ancient reptilian order Sphenodontia and the parasites in the blood have an unusual morphology. According to the illustrations in Laird, 1950 [37], three types of gametocytes may be seen: (i) bean-shaped intra-erythrocytic gametocytes of a classical type with a folded tail inside a surrounding envelope, (ii) intra-leucocytic smaller forms surrounded by an ellipsoid thick envelope which prevents full staining of the gametocyte (Fig. 3, in Laird 1950 [37], Fig. 1B, in Herbert et al. 2010 [29]), and (iii) small elongated parasites, with pointed ends (Figs. 1 and 2, in Laird 1950 [37], Fig. 1A in Herbert et al., 2010 [29] and Fig. 9 in Desser, 1978 [16]) considered by Laird to be merozoites. Herbert et al. 2010 described oocysts and sporocysts in the haemocoel of the tick. However, as there is no description of the initial stages (pairing and fertilisation) and the partial sequence (233 bp) is too small, the parasite’s identity cannot be ascertained and the sequence was not included.

The majority of sequences in the phylogenetic tree fall into one of the four groups defined below and correspond to a different genus characterised by its cycle, hosts and vectors. However, a few sequences appear in a group which does not correspond to the life history supposed by their authors. The risk of errors, when classifying a haemogregarine according to molecular data only, is that, when indispensable information on their life cycle is lacking, many species with sequences deposited in the databanks are not identified correctly at the generic level.

The main sources of errors are: (i) when, in insect eating animals or their predators, sequences derived from tissues or organs where cysts from an undetermined haemogregarine are present. For example, the sequence derived from a *Hepatozoon* in the liver of a Chiroptera [59] should be interpreted with caution. We have observed on several occasions, cysts from a haemogregarine in sections of the liver from *Miniopterus* (Fig. 3) and considered them to be cysts from a *Hepatozoon* developing in a Dipteran ingested by the bat. Haemogregarine gametocytes were never found in the blood of any bat. These cysts are probably a dead end. (ii) When the host is polyparasitised and only one of the species is sequenced. (iii) When a vector from the wild is assumed to transmit the same parasite as the one seen or sequenced from the blood of the vertebrate host. The sequences of *H. fitzimonsi* KJ702453 and KR069084, originating from blood of a turtle, group with the *Hepatozoon* clade (=*Bartazoon*). The authors show images of sporocysts in the smears of ticks which may belong to ruptured oocysts of a *Hepatozoon* or of an accumulation of sporocysts from *Hemolivia*. The same applies for sequences EU43033 and EU43034 in samples extracted from ticks engorged on reptiles (*Varanus panoptes*, *Liasis fuscus* and *Dendrolaphis pustulatus*), from Australia. No description or data on the biology is attached to show that the parasite develops in the tick. The sequences extracted from the blood of Marsupials (EU430237 and EU430238) group with the *Hepatozoon* (=*Bartazoon*) clade. The authors found ticks on the host but did not demonstrate a role of the Acari in the transmission of the parasite.

### Systematics

The type species of the genus *Hepatozoon* is *H. perniciosum*, which raises a real problem since most other species identified as *Hepatozoon* and *Hemolivia* or *Karyolysus* do not undergo the process of fertilisation by syngamy. We think that the mode of fertilisation is an important character and a fundamental part of the cycle of haemogregarines. It is at present associated with sporogony in a tick or a mite and schizogony and gametocytes in a mammal.

According to current knowledge on life cycles, terrestrial haemogregarines can be classified into four types:

Type 1: Haemogregarines of mammals transmitted by ticks and mites; fertilisation by syngamy, sporogony in one stage, with complete sporocyst development inside the oocyst envelop: genus *Hepatozoon* Miller, 1908 [51], type species: *Hepatozoon perniciosum* Miller, 1908 [51].

Type 2: Haemogregarines of reptiles transmitted so far by mites; syzygy of gamonts followed by microgametogenesis with production of a small number of gametes inside a common envelop with the macrogamete; sporogony in two stages: mature oocysts release sporokinetes which penetrate inside new cells in which the sporocysts develop. Transovarian transmission occurs. Genus: *Karyolysus* Labbé, 1894 [35], type species *K. lacertae* (Danilewsky, 1886) Reichenow, 1913 [15, 61].

Type 3: Haemogregarines of reptiles and amphibians transmitted by ticks; syzygy of gamonts followed by microgametogenesis with production of a small number of gametes inside a common envelop with the macrogamete; sporogony in two stages: mature oocysts release sporokinetes which penetrate inside new cells in which the sporocysts develop. No transovarian transmission occurs. Genus: *Hemolivia* Petit et al., 1990 [58], type species, *H. stellata* Petit et al., 1990 [58].

Type 4: Haemogregarines of reptiles, amphibians, birds and rodents transmitted by biting insects: syzygy of gamonts followed by microgametogenesis with production of a small number of gametes inside a common envelop with the macrogamete. Sporogony in one stage, complete sporocyst development inside the oocyst envelop; genus *Bartazoon* n. g. Karadjian, Chavatte and Landau, type species: *Bartazoon breinli* (Mackerras 1960) [42], n. comb. (=*Hepatozoon breinli*) of the varanid lizard.

Surprisingly, *Hemolivia* and *Karyolysus* which were considered as biologically closely related belong in fact to two different clusters, the first one with the *Bartazoon* n. g. and the second one with the *Hepatozoon* of carnivores.

### Conclusion

Classification, particularly of species into genera, aims at defining biological and morphological categories common to several species. We believe that creating a taxon for species or genera is much more helpful and less confusing than leaving parasites that are obviously different together. They may be easily synonymised if new elements are produced.

We propose (i) to reassign *Hepatozoon argantis* to the genus *Hemolivia*; (ii) the following new classification of terrestrial haemogregarines, consistent with the recent phylogenetic constructions: Haemogregarines of Type I: genus *Hepatozoon*; Haemogregarines of Type II: genus *Karyolysus*; Haemogregarines of Type III: genus *Hemolivia*; Haemogregarines of Type IV: genus *Bartazoon*.

This classification is consistent with current knowledge on biology and life cycles and with molecular data on species well identified by their life history.
